# Telepsychiatry Consultation for Primary Care Treatment of Children and Adolescents Receiving Child Protective Services in Chile: Mixed Methods Feasibility Study

**DOI:** 10.2196/25836

**Published:** 2021-07-22

**Authors:** Adrian P Mundt, Matías Irarrázaval, Pablo Martínez, Olga Fernández, Vania Martínez, Graciela Rojas

**Affiliations:** 1 Departamento de Psiquiatría y Salud Mental Hospital Clínico Universidad de Chile Santiago Chile; 2 Facultad de Medicina Universidad Diego Portales Santiago Chile; 3 Departamento de Salud Mental Ministerio de Salud Santiago Chile; 4 Millennium Institute for Depression and Personality Research (MIDAP) Santiago Chile; 5 Millennium Nucleus to Improve the Mental Health of Adolescents and Youths, Imhay Santiago Chile; 6 Unidad de Psiquiatria Infantil y del Adolescente, Departamento Psiquiatría y Salud Mental Universidad de Chile Santiago Chile; 7 Centro de Salud Reproductiva y Desarrollo Integral del Adolescente (CEMERA) Universidad de Chile Santiago Chile

**Keywords:** telemedicine, psychiatry, primary health care, child protective services

## Abstract

**Background:**

Children and adolescents living under the supervision of child protective services have complex mental health care needs. The scarcity and uneven distribution of specialized mental health teams in Chile may limit the provision and quality of care for this vulnerable population. Telepsychiatry can address such health inequities.

**Objective:**

The objective of this study was to evaluate the feasibility of a telepsychiatry consultation program for primary health care (PHC) treatment of children and adolescents living under the supervision of child protective services.

**Methods:**

We developed a telepsychiatry consultation program for two rural PHC clinics located in central Chile (Valparaíso Region) and evaluated its implementation using a mixed methods study design. The program consisted of videoconferencing mental health consultation sessions scheduled twice per month (each 90 minutes long), over a 6-month period, delivered by child and adolescent psychiatrists based in Santiago, Chile. We described the number of mental health consultation sessions, participant characteristics, perceived usefulness and acceptability, and experiences with the telepsychiatry consultation program.

**Results:**

During the 6-month study period, 15 videoconferencing mental health consultation sessions were held. The telepsychiatry consultation program assisted PHC clinicians in assigning the most adequate diagnoses and making treatment decisions on pharmacotherapy and/or psychotherapy of 11 minors with complex care needs. The intervention was perceived to be useful by PHC clinicians for improving the resolution capacity in the treatments of this patient population. Limitations such as connectivity issues were resolved in most sessions.

**Conclusions:**

The telepsychiatry consultation program was feasible and potentially useful to support PHC clinicians in the management of institutionalized children and adolescents with complex psychosocial care needs living in a poorly resourced setting. A larger scale trial should assess clinical outcomes in the patient population. Regulations and resources for this service model are needed to facilitate sustainability and large-scale implementation.

## Introduction

About half of the children and adolescents living under the supervision of child protective services (CPS) in high-income countries have mental disorders [1]. In Chile—a Latin American developing country—69% of the population living under CPS has mental disorders and complex psychosocial care needs, but the availability and uneven distribution of specialized mental health care (SMHC) in Chile limit the provision and quality of mental health care for this vulnerable population [2-4]. This prompted the implementation of an intersectoral mental health care plan aimed to improve the collaboration between CPS, primary health care (PHC), and SMHC [5].

Telepsychiatry, that is, videoconferencing to deliver mental health care, might address the shortage of SMHC by facilitating remote and timely access to quality care for underserved populations (eg, rural populations) [6-8]. Technology-based solutions increase the access to care, are acceptable by culturally diverse populations, and provide mental health outcomes comparable to in-person care [8,9]. These characteristics have also contributed to the role of telepsychiatry in emergency response to the current COVID-19 pandemic [10,11]. In Chile [12] and other countries [13-15], child and adolescent telepsychiatry services have shown promising results for the treatment of mental disorders in PHC. These initiatives have received positive feedback from health care providers serving CPS populations and reported increased use of outpatient resources for children with complex mental health care needs [16,17]. Since PHC professionals usually lack training in the treatment of complex mental health problems of CPS populations, and CPS populations lack funds for traveling to receive SMHC far from their residencies, telepsychiatry solutions are promising to improve access and quality of care.

To our knowledge, no previous study has examined the collaboration between SMHC and PHC to specifically treat CPS populations. The objective of this study was to evaluate the feasibility of a telepsychiatry consultation program (TCP) for the PHC treatment of children and adolescents living under the supervision of CPS.

## Methods

### Study Participants and Procedures

A feasibility study combining descriptive quantitative and qualitative methods was conducted in two rural PHC clinics located in the Valparaíso Region of Chile. PHC clinics were conveniently selected as local health services, and staff were open to engage with this pilot project. Four psychologists and one physician working in PHC clinics were recruited. These clinicians treated children and adolescents living under the supervision of CPS as part of their usual case load. We provided the technical infrastructure, security, and protocols needed to connect the rural PHC clinicians with the SMHC team comprising 3 child and adolescent psychiatrists who were working at the Department of Psychiatry and Mental Health of Hospital Clínico Universidad de Chile, Faculty of Medicine of Universidad de Chile, and Department of Psychiatry of Hospital El Pino, Santiago. The following measures were taken to implement the intervention: (1) securing institutional permissions to move and install technological equipment, (2) installing computers and teleconferencing devices in the intervention sites, (3) ensuring that the PHC clinicians and the SMHC teams had compatible schedules, (4) training the PHC clinicians and the SMHC teams in the technical aspects of the interventions, and (5) running connectivity tests between the study sites to pilot the intervention. This study was approved by the institutional review board at Hospital Clínico Universidad de Chile. PHC clinicians provided written informed consent and participated voluntarily at any time. Patients (ie, children and adolescents living under the supervision of CPS) were not directly involved in the feasibility testing due to complicated consent procedures required for their participation.

### Intervention

The TCP used a computer-based teleconferencing service that met communication and security standards for voice over transmission control protocol/IP. Videoconferencing mental health consultation (MHC) sessions were held between PHC clinicians and the SMHC team through a secure web-based treatment platform, that is, closed network, personalized access passwords, and disabled recording function, by using the desktop video-conferencing program Vidyo (Vidyo, Inc).

Videoconferencing MHC sessions were scheduled twice per month over a 6-month period, reserving time slots of 90 minutes each. Electronic health records (EHRs) were implemented to share patient information (eg, medical records, tests, and professional reports) between PHC clinicians and the SMHC team. EHRs were updated after each videoconferencing MHC session. Neither in-person visits nor emergency treatments were included in the study, as PHC clinicians referred such cases to local SMHC consultants. The TCP was not provided to other populations.

The TCP followed the protocol of the Chilean Ministry of Health for MHC [18], providing diagnostic assistance, management recommendations for treatment-resistant cases, and SMHC referral assessment. A protocol of the Chilean Ministry of Health defines MHC as the joint and interactive activity between SMHC and PHC teams, occurring at least once per month, to facilitate the shared continuity of care [18]. The MHC should include supervision, support and training regarding clinical cases, and clinical and administrative coordination to guarantee care continuity [18]. Before the implementation of videoconferencing MHC sessions, in-person MHC was considered as the established clinical routine.

### Measurements and Analysis

The number of videoconferencing MHC sessions held during the 6-month study period was registered and compared to in-person standard MHC sessions that were provided by local SMHC consultants in the 6 months prior to TCP implementation. The following data were collected for each videoconferencing MHC session: (1) number and types of health care providers participating, duration (in minutes) of the session, and network connection technology used; (2) clinician-rated usefulness and acceptability (assessed using six closed Likert-type items, two questions with three answer choices, and an open-ended box for comments); and (3) clinical patient information and actions taken or agreed for patient management (retrieved through inspection of the shared EHRs). The aforementioned information was analyzed using descriptive statistics.

After the 6-month study period, an open-ended, email questionnaire was used to explore the experiences of PHC clinicians and SMHC teams with the TCP (ie, satisfaction, facilitators and barriers, and recommendations). Following a grounded theory approach [19], two researchers independently coded the data using open coding. Consensus was reached through discussion and the involvement of a third researcher to ensure intersubjective consensus.

## Results

Half of the MHC sessions planned (4/8, 50%) were held in the 6 months prior to the implementation of TCP. In contrast, 15 of the 24 (63%) videoconferencing MHC sessions planned were held during the 6-month study period. Nine videoconferencing MHC sessions were cancelled due to a strike of the health care workers, incompatible schedules, and low clinical demand.

On average, videoconferencing MHC sessions were conducted every 3.7 weeks, lasted 66 minutes (range 30-100 minutes), covered 1.1 cases, and involved the participation of 2.1 PHC clinicians. The SMHC team mostly used wireless connections in about 73% (11/15) of the sessions, whereas the PHC clinicians used wired connections in 73% (11/15) of the sessions. On two occasions, participants communicated over telephone due to important and unresolved internet connectivity problems. Connectivity issues caused shortening and/or interruption of 12 videoconferencing MHC sessions.

Most cases of minors included in the TCP were girls who lived under CPS (ie, not with their relatives) due to parental neglect (Table 1). The most frequent diagnostic hypothesis was mixed behavioral and emotional disorder, reported in 5 of the 11 (45%) participants. Furthermore, psychiatric comorbidity was reported in 7 (64%) patients (ie, case load of the participating PHC professionals). The SMHC team mostly made recommendations about pharmacological schemes (n=8, 73%), psychotherapies (n=4, 36%), and psychopathological assessments (n=4, 36%).

**Table 1:** Characteristics of 11 children and adolescents living under the supervision of child protective services in rural Chile who were treated in a telepsychiatry consultation program.

Variable		Participants
Sex (female), n (%)		9 (82)
Age in years, mean (SD)		14.1 (3.1)
**Living situation, n (%)**		
	Residency without family member	5 (45)
	Residency with family member	3 (27)
	Family (nuclear or extended)	3 (27)
**Reason for living under child protective services^a^, n (%)**		
	Abandonment	1 (6)
	Sexual abuse	2 (12)
	Maltreatment	2 (12)
	Parental neglect	7 (41)
	Sexual exploitation	2 (12)
	Rape	1 (6)
	Family violence	3 (18)

^a^May include more than one reason per case.

Data collected during each videoconferencing MHC session showed that these were clinically useful to PHC clinicians (Table 2); its duration was deemed adequate in 92% of occasions; and that, after each session, interest in participating in an additional session did not decrease. Qualitative data analysis (Table 3) revealed that PHC clinicians and the SMHC team perceived the TCP as useful and that it helped to improve the quality of mental health care for minors living under the supervision of CPS. PHC clinicians expressed the need to maintain the TCP over time and stated that the intervention met their expectations regarding the methodology used and guidance on pharmacological treatments. The SMHC team emphasized that the TCP allowed them to train PHC clinicians in general aspects of mental health care, and to increase the resolution capacity of PHC clinicians. Key facilitators of the implementation were positive attitudes of the authorities of PHC, clinicians, and the SMHC team toward TCP. Incomplete patient information in EHRs was one of the main barriers to implementation, followed by technological and logistic difficulties. Recommendations made for future TCP implementations included the availability of a support technician and more guidance on psychotherapy.

**Table 2:** Perceived usefulness of a telepsychiatry consultation program, as rated by primary health care clinicians (N=8).

Item	Score^a^, mean (SD)	Range
Improved clinical understanding of the case presented	2.6 (0.5)	2-3
Learning experience applicable to similar cases	2.5 (0.5)	2-3
Guidance about medications and dosage	2.6 (0.5)	2-3
Orientation about psychosocial interventions	2.5 (0.7)	1-3
Benefit for the children and adolescents	2.4 (0.6)	1-3
Benefit for the primary health clinician	2.8 (0.4)	2-3

^a^Scores range from 0 to 3, with higher scores reflecting higher perceived usefulness.

**Table 3:** Synthesis of the main categories found in the qualitative data.

Category and subcategory			Illustrative quote
Usefulness of the intervention and improved quality of care			“[Did the TCP help?] *Certainly, all the consultations and especially the last one... the person in charge of the team stated how useful the telepsychiatry consultations had been*” [Psychiatrist #1]
Need for continued support			“*It would be very useful to keep this program available, for more months”* [Primary health care psychologist #4] “…*It would be good for it to be a regular program, it would greatly contribute to mental health teams in primary care”* [Primary health care psychologist #3]
**Meeting expectations**			
	On the methodology used	“*There was a clear methodology and a well-defined way of handling each case”* [Primary health care physician #1] “*The clinical advice enabled us to develop teamwork, which added dynamism to the care we delivered, …communication became more effective, and that strengthened teamwork”* [Primary health care psychologist #3]	
	On guiding pharmacological treatments	“*The goal of the consultations was met, especially regarding the clarification of doubts about the pharmacotherapy”* [Psychiatrist #1]	
Training primary health care practitioners and increased resolution capacity			“*It not only enabled us to give them advice about specific cases; we also trained them about more general aspects. I sent them complementary material for psychotherapy approaches”* [Psychiatrist #3] “*I specifically helped them by increasing their self-confidence and facilitating their decision-making processes”* [Psychiatrist #1]
**Facilitators and barriers to implementation**			
	Facilitators to implementation	“*Openness of the directors, motivation of staff members”* [Primary health care psychologist #1] “*The willingness to help of the psychiatrist*” [Primary health care psychologist #4] “*Punctuality, willingness to participate and interest in the activity, they were open to my suggestions and advice as a consultant”* [Psychiatrist #1] “*I enjoy teamwork and have a personal interest in telepsychiatry”* [Psychiatrist #2]	
	Barriers to implementation	“[On electronic health records] *They don't include all the information that we'd need for an adequate teleconsultation session, for example, the medications that the patients were taking”* [Psychiatrist #3] “*The [residence] does not have full clinical records…about the patient”* [Primary health care physician #1] “*Interruptions due to technical problems such as internet access, availability of an office and/or computer”* [Psychiatrist #1] “*Sometimes we had no access to a computer and a suitable room…, which delayed the process”* [Primary health care psychologist #1]	

## Discussion

### Main Findings

The implementation of TCP was feasible and supported PHC clinicians in diagnosing and/or treating children and adolescents with complex psychosocial care needs living under the supervision of CPS. The intervention was perceived to be clinically useful for the patient population, and it increased the resolution capacity of PHC clinicians and their willingness to receive a future TCP.

### Strengths and Limitations

To our knowledge, this is the first study to report a TCP connecting SMHC teams with PHC clinicians to improve mental health care among minors living under the supervision of CPS. A protocol for future implementations was developed. To improve the generalizability of the study results, larger and geographically more varied samples are needed to reflect the heterogeneous scenario of PHC and CPS in resource-poor Latin American contexts. A limitation of the study was that it did not directly assess mental health outcomes in the minors living under the supervision CPS. The common connectivity issues experienced during the study period were a further limitation, although they were fixed in a timely manner and usually did not affect the workflow of the MHC. Nevertheless, they may be important barriers to the adoption of TCPs in resource-constrained rural areas. Other important limitations of the implementation process included the lack of patient information and history in the EHRs, reflecting problems of sharing essential information among CPS, PHC, and SMHC.

### Comparison With Literature

In Chile, the use of technology-based solutions for mental health care of remote and underserved populations has been important due to the geography, and it has become increasingly relevant during the COVID-19 pandemic—with a rapid shift to this mode of mental health service delivery to ensure access and continuity of care [11]. For instance, partnerships between centralized SMHC teams and PHC clinicians through the implementation of call centers and EHRs have been reported to be acceptable and satisfactory for PHC clinicians as well as adults and adolescents with depression [12,20]. This study is in line with these experiences and provides initial evidence for the feasibility of telepsychiatry—in the form of videoconferencing MHC and EHRs—to support PHC clinicians in the mental health care of children and adolescents with complex psychosocial care needs.

Evidence from the United States has provided strong foundations for the feasibility and potential efficacy of community-based child and adolescent telepsychiatry [13-15]. Digital health interventions, including videoconferencing MHC, have also been found to be satisfactory and acceptable for health care providers, caregivers, and youths in juvenile community-based justice settings [21-23]. Many of these youths came from CPS and have important behavioral health needs, yet telepsychiatry interventions specifically aimed at addressing the needs of CPS population and their providers in community settings are lacking.

Experiences reported from the United States resemble those reported in the present study in that they have applied technology-based solutions to build partnerships between SMHC and community or PHC providers, having included data from CPS population in their analyses [16,17]. These statewide initiatives in Washington [16] and Wyoming [17] considered centralized child psychiatric telephone and videoconferencing MHCs for health care providers of the population covered by Medicaid. In these studies, MHC were initiated by community or PHC providers in need of guidance [16,17]. These studies demonstrated higher satisfaction among those providers treating institutionalized children and adolescents, significant declines in high-dose pediatric psychotropic prescribing, as well as an increase in the use of outpatient and community-based treatments among this population.

Although our findings are consistent with these experiences [16,17], this feasibility study was specifically aimed at supporting the PHC treatment of children and adolescents living under the supervision of CPS. The TCP provided a fixed schedule for the linkage between SMHC teams and PHC clinicians within the context of a specific intersectoral mental health care plan, including CPS, PHC, and SMHC. These intersectoral alliances create an opportunity for further implementation, evaluation, and refinement of telepsychiatry.

### Concluding Remarks

Implementing a TCP was feasible and potentially useful for addressing mental health challenges in PHC of children and adolescents living under the supervision of CPS. Future programs may consider incorporating a child and adolescent psychologist to the SMHC team. To demonstrate the effectiveness of these interventions, a future clinical trial should assess the clinical outcomes in the children and adolescents living under the supervision of CPS. Furthermore, future research should consider scaling the TCP to more PHC clinics, including a larger patient population. Health care policies should be developed to provide regulation and resources for this model of mental health care, to be sustainable over time.

## Abbreviations

CPS: child protective services
EHR: electronic health record
MHC: mental health consultation
PHC: primary health care
SMHC: specialized mental health care
TCP: telepsychiatry consultation program

## References

[1] Bronsard G, Alessandrini M, Fond G, Loundou A, Auquier P, Tordjman S, Boyer L (2016). The prevalence of mental disorders among children and adolescents in the child welfare system: a systematic review and meta-analysis. Medicine (Baltimore).

[2] Comité de los Derechos del Niño (2018). Informe de la investigación relacionada en Chile en virtud del artículo 13 del Protocolo facultativo de la Convención sobre los Derechos del Niño relativo a un procedimiento de comunicaciones. Webpage in Spanish.

[3] Minoletti A, Alvarado R, Rayo X, Minoletti M (2014). Evaluación del sistema de salud mental en Chile. Informe sobre la base del Instrumento de evaluación del sistema de salud mental de OMS (OMS IESM/WHO AIMS). Report in Spanish.

[4] Saldivia S, Vicente B, Kohn R, Rioseco P, Torres S (2004). Use of mental health services in Chile. Psychiatr Serv.

[5] Valenzuela Azócar C, Santander Cortéz X, Véliz Rojas V, Cordero Rojas A, De Ferrari Fontecilla I (2018). Informe de Gestión SENAME de la Subsecretaría de Redes Asistenciales Años 2016-2018. Report in Spanish.

[6] Gardner J, Plaven BE, Yellowlees P, Shore J (2020). Remote telepsychiatry workforce: a solution to psychiatry's workforce issues. Curr Psychiatry Rep.

[7] Myers CR (2019). Using telehealth to remediate rural mental health and healthcare disparities. Issues Ment Health Nurs.

[8] Hilty DM, Sunderji N, Suo S, Chan S, McCarron RM (2018). Telepsychiatry and other technologies for integrated care: evidence base, best practice models and competencies. Int Rev Psychiatry.

[9] Hilty DM, Rabinowitz T, McCarron RM, Katzelnick DJ, Chang T, Bauer AM, Fortney J (2018). An update on telepsychiatry and how it can leverage collaborative, stepped, and integrated services to primary care. Psychosomatics.

[10] Kinoshita S, Cortright K, Crawford A, Mizuno Y, Yoshida K, Hilty D, Guinart D, Torous J, Correll CU, Castle DJ, Rocha D, Yang Y, Xiang Y, Kølbæk P, Dines D, ElShami M, Jain P, Kallivayalil R, Solmi M, Favaro A, Veronese N, Seedat S, Shin S, Salazar de Pablo G, Chang C, Su K, Karas H, Kane JM, Yellowlees P, Kishimoto T (2020). Changes in telepsychiatry regulations during the COVID-19 pandemic: 17 countries and regions' approaches to an evolving healthcare landscape. Psychol Med.

[11] Fischman P, Irarrazaval M (2020). Debate: Mental health, social crisis and the COVID-19 pandemic in Chile. Child Adolesc Ment Health.

[12] Martínez V, Rojas G, Martínez P, Zitko P, Irarrázaval M, Luttges C, Araya R (2018). Remote collaborative depression care program for adolescents in Araucanía Region, Chile: randomized controlled trial. J Med Internet Res.

[13] American Academy of Child Adolescent Psychiatry (AACAP) Committee on Telepsychiatry and AACAP Committee on Quality Issues (2017). Clinical Update: Telepsychiatry With Children and Adolescents. J Am Acad Child Adolesc Psychiatry.

[14] Myers K, Nelson E, Rabinowitz T, Hilty D, Baker D, Barnwell SS, Boyce G, Bufka LF, Cain S, Chui L, Comer JS, Cradock C, Goldstein F, Johnston B, Krupinski E, Lo K, Luxton DD, McSwain SD, McWilliams J, North S, Ostrowsky Jay, Pignatiello A, Roth D, Shore J, Turvey C, Varrell JR, Wright S, Bernard J (2017). American telemedicine association practice guidelines for telemental health with children and adolescents. Telemed J E Health.

[15] Marcus S, Malas N, Dopp R, Quigley J, Kramer AC, Tengelitsch E, Patel PD (2019). The Michigan Child Collaborative Care Program: building a telepsychiatry consultation service. Psychiatr Serv.

[16] Hilt R, Romaire MA, McDonell M, Sears J, Krupski A, Thompson J, Myers J, Trupin EW (2013). The Partnership Access Line: evaluating a child psychiatry consult program in Washington State. JAMA Pediatr.

[17] Hilt R, Barclay R, Bush J, Stout B, Anderson N, Wignall J (2015). A statewide child telepsychiatry consult system yields desired health system changes and savings. Telemed J E Health.

[18] (2016). Orientaciones Técnicas: Consultorías en Salud Mental. Report in Spanish.

[19] Strauss A, Corbin J (2016). Bases de la investigación cualitativa. Técnicas y procedimientos para desarrollar la teoría fundamentada. Book in Spanish.

[20] Rojas G, Guajardo V, Martínez Pablo, Castro A, Fritsch R, Moessner M, Bauer S (2018). A remote collaborative care program for patients with depression living in rural areas: open-label trial. J Med Internet Res.

[21] Batastini Ashley B (2016). Improving rehabilitative efforts for juvenile offenders through the use of telemental healthcare. J Child Adolesc Psychopharmacol.

[22] Tolou-Shams Marina, Yonek Juliet, Galbraith K, Bath E (2019). Text messaging to enhance behavioral health treatment engagement among justice-involved youth: qualitative and user testing study. JMIR Mhealth Uhealth.

[23] Folk J, Harrison A, Rodriguez C, Wallace A, Tolou-Shams M (2020). Feasibility of social media-based recruitment and perceived acceptability of digital health interventions for caregivers of justice-involved youth: mixed methods study. J Med Internet Res.

